# Carbon nanotubes: a novel innovation as food supplements and biosensing for food safety

**DOI:** 10.3389/fnut.2024.1381179

**Published:** 2024-05-13

**Authors:** Maazallah Masood, Tala Albayouk, Na'il Saleh, Mohamed El-Shazly, Heba A. S. El-Nashar

**Affiliations:** ^1^Department of Biotechnology, International Islamic University, Islamabad, Pakistan; ^2^Department of Chemistry, College of Science, United Arab Emirates University, Al Ain, United Arab Emirates; ^3^Department of Pharmacognosy, Faculty of Pharmacy, Ain Shams University, Abbassia, Cairo, Egypt

**Keywords:** carbon nanotubes, food industry, nanosensors, quality control, food safety

## Abstract

Recently, nanotechnology has emerged as an extensively growing field. Several important fabricated products including Carbon nanotubes (CNTs) are of great importance and hold significance in several industrial sectors, mainly food industry. Recent developments have come up with methodologies for the prevention of health complications like lack of adequate nutrition in our diet. This review delves deeper into the details of the food supplementation techniques and how CNTs function in this regard. This review includes the challenges in using CNTs for food applications and their future prospects in the industry. Food shortage has become a global issue and limiting food resources put an additional burden on the farmers for growing crops. Apart from quantity, quality should also be taken into consideration and new ways should be developed for increasing nutritional value of food items. Food supplementation has several complications due to the biologically active compounds and reaction in the *in vivo* environment, CNTs can play a crucial role in countering this problem through the supplementation of food by various processes including; nanoencapsulation and nanobiofortification thus stimulating crop growth and seed germination rates. CNTs also hold a key position in biosensing and diagnostic application for either the quality control of the food supplements or the detection of contagions like toxins, chemicals, dyes, pesticides, pathogens, additives, and preservatives. Detection such pathogens can help in attaining global food security goal and better production and provision of food resources. The data used in the current review was collected up to date as of March 31, 2024 and contains the best of our knowledge. Data collection was performed from various reliable and authentic literatures comprising PubMed database, Springer Link, Scopus, Wiley Online, Web of Science, ScienceDirect, and Google Scholar. Research related to commercially available CNTs has been added for the readers seeking additional information on the use of CNTs in various economic sectors.

## Graphical Abstract

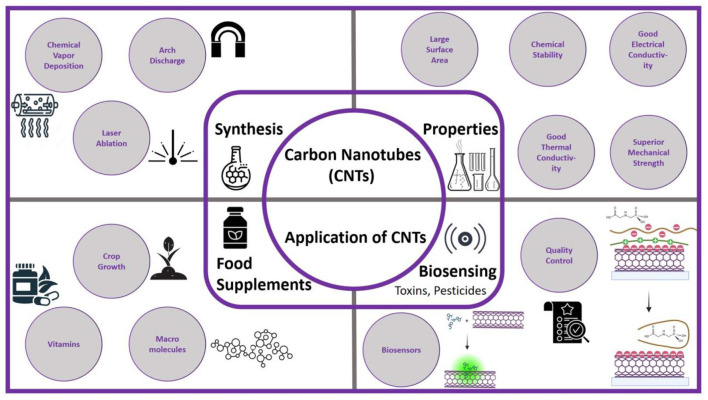

## 1 Introduction

Carbon nanotubes (CNTs) are comprised of coiled up concentric graphene sheets (1). They have a diverse range of applications due to their unique morphology which confers various useful properties including optical activity, thermal conductivity and electrical signaling (2). These attributes lead to their use in various mechanical applications as well and widen their prospective use in different industrial sectors (3, 4). Owing to their useful properties, CNTs have been used in various sectors including biomedical industry (5) for medicines delivery (6), treatment of diseases (7, 8) and diagnosis of various ailments (9–11). This review focuses specifically on the food sector where CNTs are not only used as supplements but also play a major role in quality control of food products with the help of efficient nanobiosensing mechanisms (12–14). CNTs are classified on the basis of their morphology and mode of synthesis. They can be stratified into various classes based on the number of walls present within their structure. Based on their morphological characteristics, they are grouped into three major categories; (a) Single walled carbon nanotubes (SWCNTs), (b) Double walled nanotubes (DWCNTs), and (c) Multi walled carbon nanotubes (MWCNTs) respectively. The term SWNTs was first described in 1993 (15) and they are named so, due to a single layer of atomic graphite that is present in their structure. These are arranged in a cylindrical fashion in the CNT architecture and range from 1 to 2 nm in size. This small size confers useful properties to them and allows them to be utilized in a wide range of applications including electrical and mechanical domains (16). The second type of CNTs, known as DWCNTs are composed of two carbon nanotubes which are distinct from one another. The basic morphology of DWCNTs includes an inner tube present within the external tube and the surrounding tube having a diameter of 2–4 nm while the internal one is about 1–3 nm in width (17, 18). The third type of CNTs are known as MWCNTs. These are characterized by a number of graphene sheets, which roll up to make a diameter of about 2–50 nm. The radius of the inner tube is 0.34 nm to be precise while the outer layer has a radius ranging from 20 to 30 nm. Two different models are also present for structural studies of CNTs, they are known as Parchment and Russian doll models. In the former model, single graphene sheets are rolled up upon themselves while in the latter case, sheets of graphene are stacked in the form of coordinated cylinders. MWCNTs are composed in a form of meshwork with combinations of SWCNTs having varying diameters (16). Owing to their useful structural insights, CNTs find useful roles in the food industry where they have been employed for flavor enhancement, controlled release of nutrients and enhancement of bioavailability of nutritionally important substances. Novel properties of CNTs, especially nano encapsulation, has been implied in various researches in an effort to increase nutritional value of various food items. The controlled release of nutrients would not only provide important nutrients to the consumer but also check the required amount that one needs for daily consumption. This can prove to be a breakthrough for nutritionists in determining a balanced diet and CNTs can play a major role in this phenomenon. Apart from this, CNTs can be used to provide food supplements by nano fortification process and enhance seed characteristics and nutritional importance. The major benefit of using CNTs in agriculture can prove to be a hallmark of biotechnology owing to food quality problems worldwide. Many researchers have studied the potential of CNTs in enhancing germination and quality without any toxic side effects (19, 20). The foregoing account makes it clear that CNTs can play an important role in the food industry in the future due to their unique properties at micro scale. Apart from the food industry and supplements, CNTs are being investigated as bio sensing materials and have been employed in the synthesis of commercially available products as evident from Table 2. Toxins can contaminate food items and render it misfit for consumption. CNTs provide a useful role in the detection of toxins in food items where they can play key roles in attaining food safety goals, worldwide. CNTs have also been employed in the detection of pesticides and pathogens as both create problems during food consumption (21). Novel techniques including High Performance Liquid Chromatography (HPLC), Liquid Chromatography (LC) and Gas Chromatography (GC) have been used in studying and detecting harmful substances. CNTs have also be used in the detection of food preservatives and dyes which can be harmful for health (22–24). All the afore-mentioned traits and applications of CNTs exhibit their useful role in the food industry and to be used as biosensing materials in the future.

CNTs possess thermal, mechanical and electrical properties which are a consequence of their unique structure and aspect ratio (25). Carbon–carbon sp^2^ bonding is responsible for stiffness and axial strength of CNTs. Electrical properties of CNTs can be attributed to their one directional nature and electronic structure of graphite. One of the most important properties is their low electrical resistance that confers useful properties to CNTs. They exhibit the highest current density as compared to any other substance with values as high as 10^9^ Acm^−2^. CNTs also possess the ability to conduct current at lower temperatures, a property that is not shown by conventional conductors. These properties have useful applications in fabricating field emission displays (FEDs) and electronic computer circuits.

**Figure 1:**
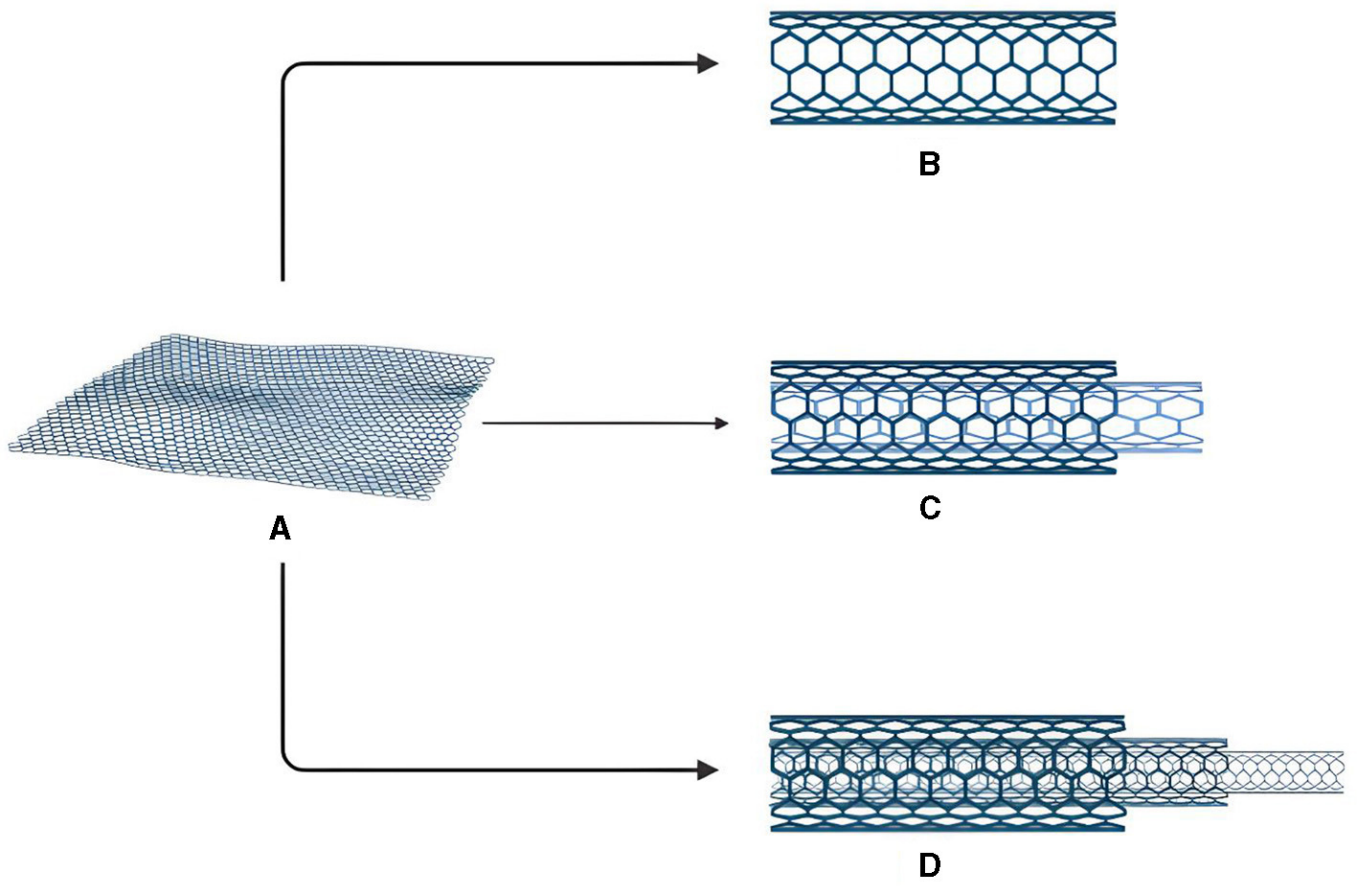
Structure and types of various CNTs. **(A)** Graphene sheet **(B)** SWCNTs **(C)** DWCNTs **(D)** MWCNTs.

CNTs are known to be better conductors than diamonds, which is known to possess best thermal conductivity parameters. CNTs are twice as good as diamonds in this regard. CNTs are chemically inert, which makes them biologically and chemically stable and compatible. Surface to volume ratio being higher is being studied for storage purposes for gases including hydrogen. SWCNTs are also employed as vectors for drug delivery, carrying the materials across the cells and their small diameter confers useful properties to them. One of the key properties is their attachment to normal atomic force microscopy tips and improvement of resolution and image quality. Specific one pair receptor/target binding studies are made much easier by utilizing the nanoscale architecture of SWCNTs. SWCNTs possess electronic abilities and are capable of extracting electrons due to which visible signals are observed in the form of light ranging from visible to X-ray region. This becomes the bases of various biosensing applications and detection of target materials (17). CNTs are also capable of specifically binding energetic materials to their target sites and keeping them active for extended periods of time (19). The general structure of CNTs and their types can be seen in Figure 1.

## 2 Manufacturing and characterization of CNTs

### 2.1 Methods of manufacturing CNTs

Various methods are used for the manufacturing of CNTs and three of them have been mentioned in this current review. The first one is known as arc discharge (AD) method. It is the most conventional method in which an arc is produced by direct current using two electrodes. Carbon rods are set in an inert chamber with a gas (26). The gas itself is provided at a steady rate that permits fast accumulation of carbon at the cathode. The deposition process is further enhanced by the presence of plasma in the chamber. Two significant parameters govern the method of synthesis, the first one is the controlled arc current, and the second one is the inert gas pressure. The mode of action of this process includes keeping the pressure of the non-reactive gas consistent in the chamber and steady supply of electric current. It results in the positive electrode to be drawn closer to the negative terminal and it results in the production of an arc. This process results in a rise in temperature to such as extent that plasma is formed, and electrodes become intensely hot. CNTs are then deposited at the negative electrodes and thus this procedure can be used for the synthesis of SWCNTs and MWCNTs. CNTs produced by this method have a diameter of ~2–20 nm respectively (27). The conditions required for synthesis include a voltage of 25–60 V, current being 100A and graphite as the carbon source (28).

The second method employed for the manufacturing of CNTs is known as laser ablation (LA). In this method, a laser beam is utilized to vaporize carbon (29). The graphene surface is heated via laser and the graphene rods themselves are composed of cobalt and nickel which are subjected in an oven having a temperature of about 1,200°C in an inert atmosphere. Bigger particles are separated with the help of two pulses to produce CNTs. CO_2_ laser can be utilized for the delivery of SWCNTs while another important parameter found to be the deciding factor for increased CNT diameter, is the power of the laser employed for synthetic process. This method is different from the previous ones as it doesn't use heat above 1,200°C, pressure up to 500 torr and usage of graphite.

**Figure 2:**
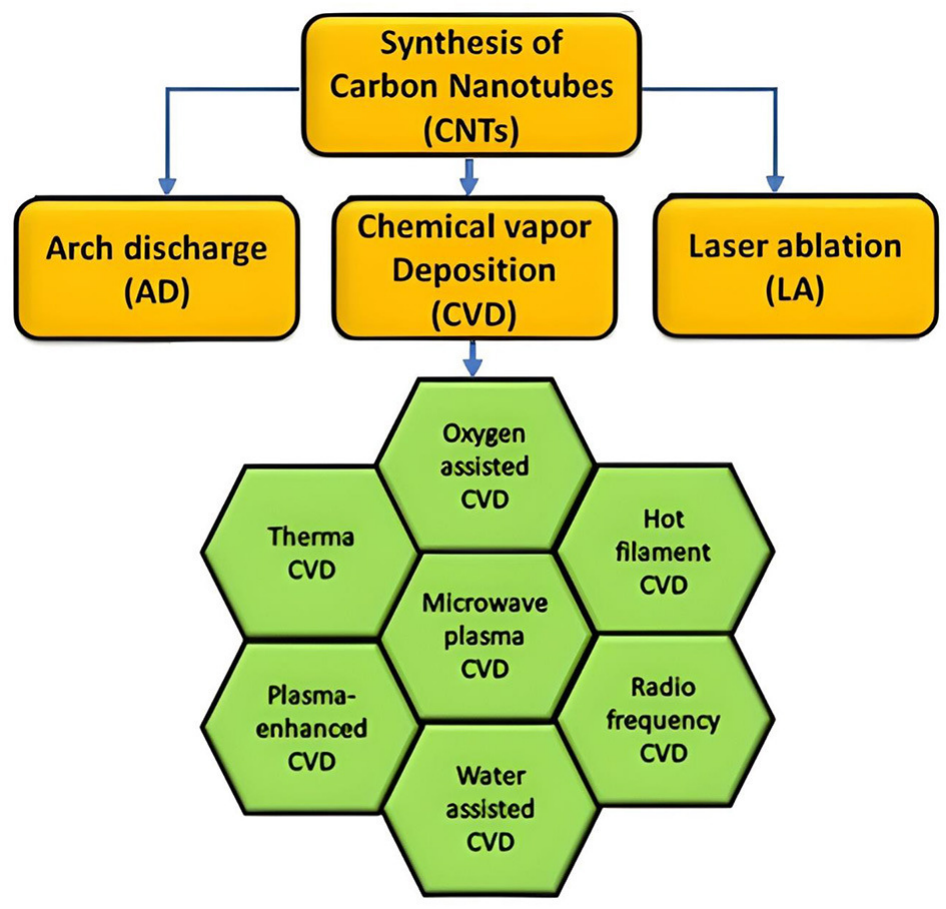
Methods of fabricating CNTs.

The third technique to manufacture CNTs is known as Chemical Vapor Deposition (CVD) method. This technique uses a carbon source along with a catalyst and produces CNTs having irregular and dispersed graphene sheaths (30). CVD uses hydrogen gas and other processed gases such as N_2_ and H_2_. The gas decomposition process causes CNTs to act as catalysts and be grown on the substrate. These are then implanted with nanoparticles including Ni, Co, and Fe. The hydrocarbon deposits on the substrate and decomposes, it is then connected to the metal particles generating CNTs later on (31–33). Apart from these three methods, flame synthesis, gas phase catalytic (HiPCO), aerosol precursor method, core shell polymer, arc water process, microsphere method plasma method, low temperature route, nebulized spray and fluidized bed method can also be utilized for manufacturing CNTs for a number of applications (17). The techniques and different methods of fabricating CNTs are exhibited in Figure 2.

### 2.2 Functionalization of CNTs

**Figure 3:**
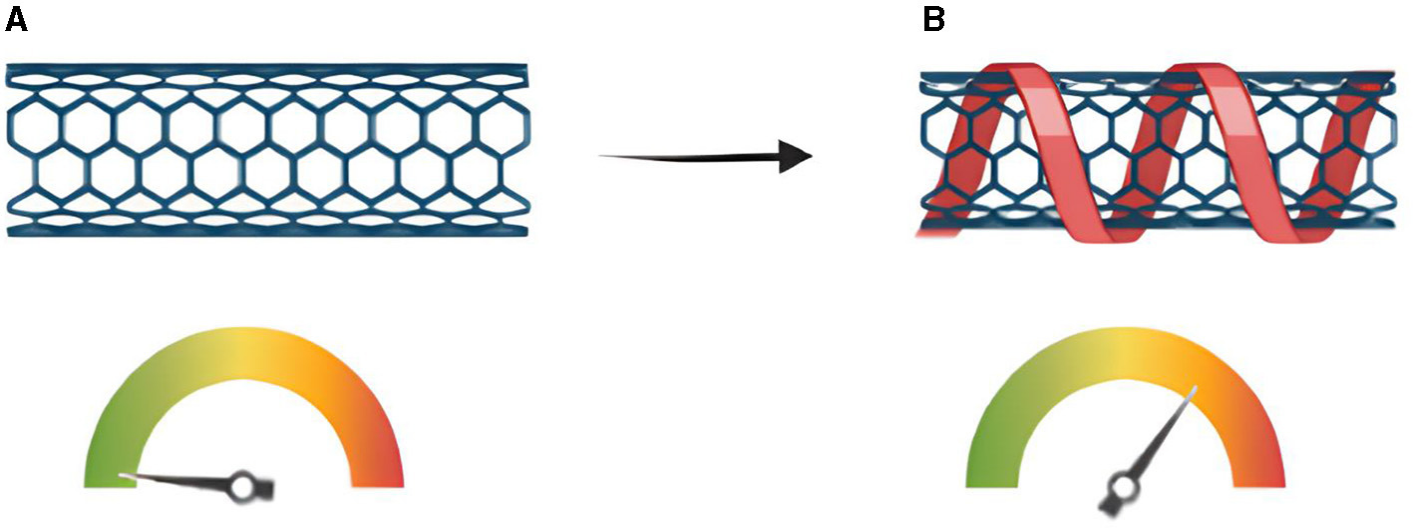
Mechanism of working **(A)** CNT with no immobilization so that the conductivity rate stays the same **(B)** CNT as some molecules immobilized on its surface and cause the conductivity to change.

Functionalization of CNTs can be performed by utilizing two processes; (a) Covalent bonding and (b) Noncovalent (van der Waals bonds). The former includes functional groups (COOH, OH, and NH_2_) attached to sp^2^ carbon structure. These functional groups are adhered to carbon nanotubes and result in better solubility parameters and disruption. The latter includes Van der Waals and π–π interactions. Surface modification can be performed with noncovalent bonds with surfactants, sidewall functionalization and noncovalent hexahedral polymers respectively. These modifications provide useful properties to CNTs including thermodynamic and interactive traits. The sidewall functionalization includes sp^2^ to sp^3^ interaction with the help of nucleophilic attack on aromatic rings. The noncovalent functionalization is categorized by the presence of amphiphilic entities including surfactants (34). These molecules possess hydrophilic and hydrophobic portions and are capable of reducing surface tension among two immiscible liquids. The use of surfactants with CNTs results in better dispersion properties and functionality of CNTs. Endohedral functionalization involves the inner space modification of CNTs. The inner space of CNTs can be modified with the help of various mechanisms and thus the space is considered as a nano reactor environment that can be modified for useful applications and properties. External surface can also be modified in an approach known as exohedral functionalization in which functional groups can be attached to the surface of CNTs to enhance their properties and use in various applications (34). The mechanism of working of CNTs and its relation to conductivity is illustrated in Figure 3.

## 3 Role of CNTs in the food industry

CNTs find novel applications in food safety, detection of purity, quality and quantification of toxins, chemicals, pesticides, pathogens, and food dyes. However, they also play a key role as mediators of supplementation in food via different techniques as discussed below.

### 3.1 Nano food supplements and crop growth enhancement

Food supplements are important in the regulation of health and are considered as driving nutritional factors toward a sound life. They are incorporated into the diet to prevent deficiencies by bridging the gap between diet and essential nutrients (35). Severe diseases and health issues such as cardiovascular disorders, anemia, and carcinogenesis can arise due to the disruption of metabolic pathways *in vivo*, due to the lack of nutrition in diet, comprising of vitamins and essential supplementary ingredients such as antioxidants. The supplementary food ingredients are degraded during processing steps and need to be made a part of food again. A solution to this problem is tackled by nanotechnology which improves food quality with the help of several mechanisms. One such mechanism is known as Nanoencapsulation (NE) that has been procured specially because of its distinctive features for efficient nano-scale encapsulation, enhanced stability, and better-controlled release of encapsulated materials. NE is a highly replicable methodology as it conserves the flavor of food items. Various food supplements have been processed using this novel strategy (36). Details of some encapsulated materials can be found in the following text.

**Figure 4:**
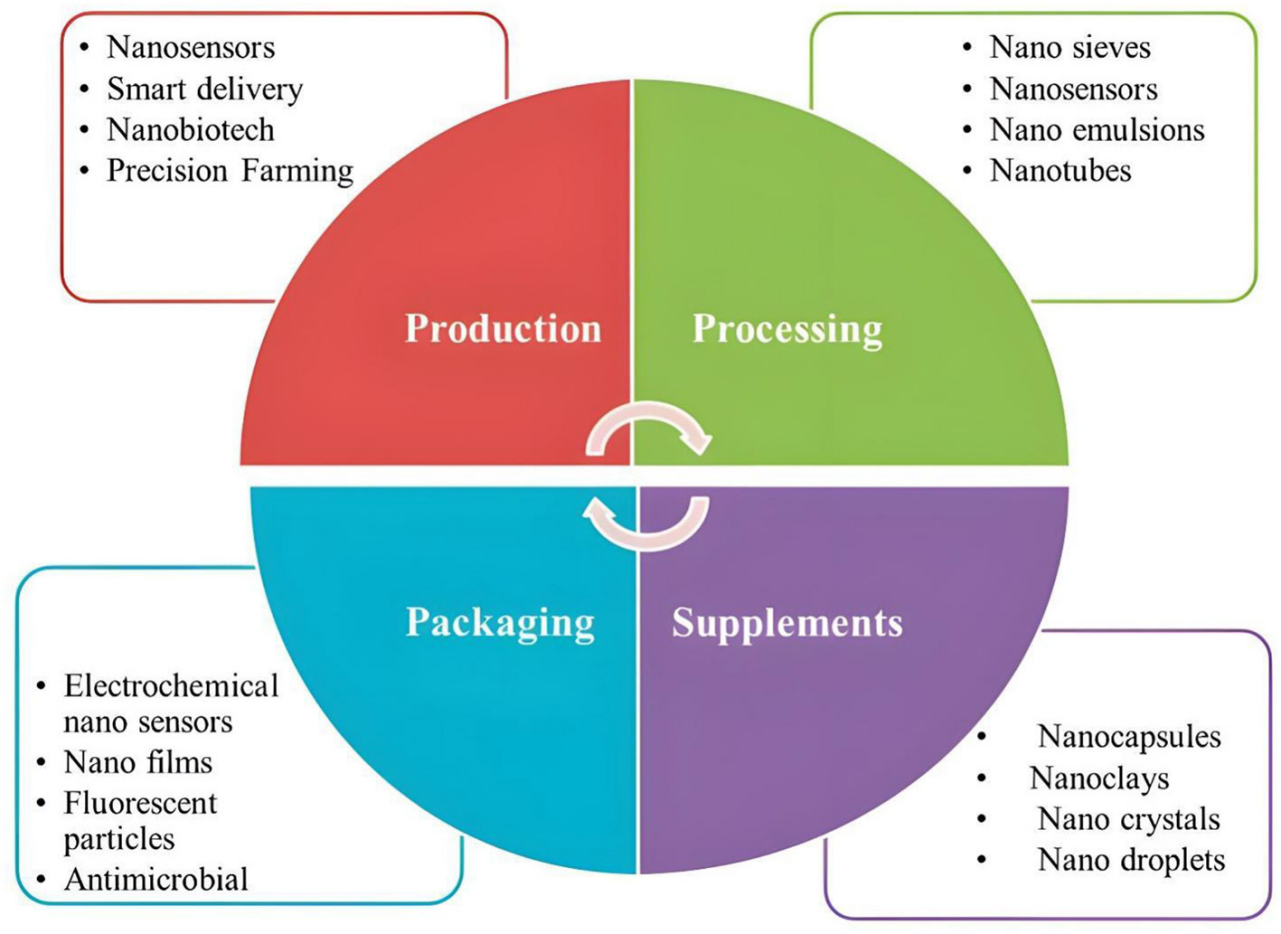
Role of CNTs in food industry (19). The figure is a part of free to use, creative commons license and requires no copyright permission.

Anthocyanins are nano encapsulated due to being extremely reactive. Nanoencapsulation of cyanidin-3-0-glucoside within the center cavity of a modified kernel of Glycine max enhances the stability of the kernel via the H2 subunit of ferritin (rH2) whereas rutin (a dietary flavonoid), is used less in the food sector due to its instability. However, NE of ferritin nanocages enhanced the physical properties of rutin in contrast to non-NE. Usually, increasing the bioavailability of the biologically active compounds is much more common by nanoemulsions. In addition, biologically active compounds like carbohydrates, lipids, vitamins, and proteins are susceptible to degradation by the enzymes that are active in the acidic environment of the stomach and duodenum. Their lower stability is owed to their activity and less solubility traits. Countering this problem, nanoencapsulation allows these biologically active compounds to withstand these adverse factors thus making them more soluble, tougher, and stable. Daily foods may consist of nanomaterials immobilized on small edible capsules for the provision of health benefits and the efficient delivery of important micronutrients including some vitamins (19). Nano-fortification can also be performed with the help of CNTs and carbon NPs (37). The properties of nanomaterials like MWCNTs and SWCNTs alter at the nanoscale as compared to their bulk counterparts due to which they are occupied in crop production. CNTs usually don't dissolve in aqueous media due to their high hydrophobicity but they have the capacity to form van der Waals forces. Specifically, SWCNTs have a hydrophilic nature and are large in size due to which they can't cross the cell walls of plants. Thus, MWCNTs can cross the cell wall barrier and provide useful properties regarding quality of food products. Seed germination is enhanced by the incorporation of MWCNTs in *Solanum lycopersicum* where MWCNTs show no toxicity toward crops including *Lactuca sativa, Cucumis sativus, Triticum*, and *Zea mays*. Research shows that long-term exposures didn't exhibit toxicity problems, however the positive impact was seen as observed by Lahiani et al. on the growth of crops. Lahiani concluded that different concentrations of CNTs act as different stimulus for plant growth. In case of *S. lycopersicum*, a lesser concentration (25 μg/ml) gave better germination results as compared to 100 μg/ml treatment. In addition, magnetic NPs can be used to aid the process as the chemicals are protected and nanomaterials are directed inside the structure of the plants (38). Nile reported that SWCNTs are utilized in the food industry for the manufacturing of honey and wine. Patel found out that a low concentration of MWCNTs enhances the growth of mustard seeds and increases the rate of germination for *S. lycopersicum* seeds respectively. In addition, it was reported that the rate of root growth was enhanced by non-functionalized CNTs in *C. sativus, Z. mays*, and *Allium cepa* respectively (28). Thus, nanomaterials aid the nutrition at the nanoscale and can help us to evade different complications related to food processing and growth. Additionally, the accumulation of cholesterol can be prevented by nutraceuticals possessing phytosterols, β-carotenes, and lycopene and lead to better quality parameters (39). Figure 4 shows the role of CNTs and their applications in the food industry.

### 3.2 Quality control for supplements

The detection of numerous deteriorated supplements is extremely crucial for ensuring better health and that is why the utilization of various nanosensors and nanostructures are coming into play. Supplements such as folic acid (FA) and ascorbic acid (AA) can be detected in several fruit juices, *Triticum* flour and milk samples by nanostructure based CNTs. The number of degraded supplements can be calculated using CNTs and it can be made sure if the basic requirements and the minimum quantities of the supplementary ingredients are being fulfilled in the diet or not. FA plays a vital role in the prevention of complications in pregnancy such that its deficiency can lead to fetal abnormalities during development in the neural tube. In addition, FA is not synthesized in the human body due to which a minimum dose having 400 μg of synthetic FA needs be daily incorporated in the diet by pregnant women especially as it is essential for fetal growth and development (40). Unstable key food ingredients like folate and vitamin B9, H_2_O_2_ and tryptophan can also be detected by PtCb SWCNT, DWCNT and MWCNT by amperometry and cyclic voltammetry techniques (19). In a research, efficient identification and detection of bacterial cells without labeling, SWNT-mediated potentiometric aptamer biosensor was implemented in milk having 6 cfu/ml and malus juice having 26 cfu/ml respectively (3). Quality control is a crucial step in food safety and processing as it ensures that supplements do not exceed their normal values and it can lead to dangerous health implications and complications, thus deteriorating the quality of life (41).

## 4 Role of CNTs in biosensing for food safety

Biosensing has been enhanced in the last couple of years and it has really picked up the pace due to the CNTs based biosensing designs which enhance the efficacy of food analysis. The large area to surface ratio owing to their nano size, rapid response rate in the form of luminescence or electrochemical signals along with stability and higher sensitivity lead to an increased use of CNTs in biosensing applications (28, 42, 43).

The working principle behind biosensing using carbon nanotubes consists of CNTs being used as scaffolds for biomolecules that can be immobilized on their surface (44–46). Phenomenon including physical, chemical, and optical properties makes them the best choice for biosensing materials. The basic mechanism of action includes the changes in electrical signals associated with recognition of metabolites and analytes. CNTs are known to conduct electricity and hence serve as indicators of various substances that can attach to their surface. The adherence of materials leads to the production of electrical signals that cause changes in CNTs and help in biosensing phenomenon (47, 48). The following Table 1 shows some contagions related to food and their implications on human health.

**Table 1:** Contaminants with their effects on human health.

**Sr. No**.	**Name of contaminants**	**Nature of contaminants**	**Implications on human health**	**References**
1	(Roundup) glyphosate, N-(phosphonomethyl) glycine	Glyphosate based herbicide	Effects the regulators of human reproductive system	(49, 50)
2	Azoxystrobin	Fungicide	Cause of cell death and neurotoxin	(51, 52)
3	Neonicotinoids	Insecticide	Genetic and birth defects of cardiovascular, respiratory and neurological systems	(53)
4	Atrazine	Herbicide	Low fetal weight, cancer, and muscle spasms	(54, 55)
5	Chloropyrifos	Insecticide	Autoimmune and development disorders	(56, 57)
6	Lead, arsenic, mercury, cadmium	Heavy metals	High blood pressure, carcinogenic effect, neurological disorders and renal failure	(58)
7	*Staphylococcal, Enterotoxins, Salmonella*	Pathogens, microorganisms	Food poisoning and illness	(59, 60)
8	Food coloring Blue 1 and 2, Yellow 5 and 6, Citrus Red 3, Red 2, Green 3 and Red 40, Tartrazine, Sunset yellow, Erythrosine	Food dyes	Link with autism, depression, hyperactivity, eczema, lupus, migraines, infertility, asthma, thyroid cancer, and genotoxicity	(61–66)
9	Sodium benzoate	Preservatives, additives	Neurodevelopmental behavioral disorders	(67, 68)
10	Aflatoxin, fumonisins	Toxins, mycotoxins	Defect in immune system, birth defects and cancer	(69–71)

### 4.1 Toxins

**Figure 5:**
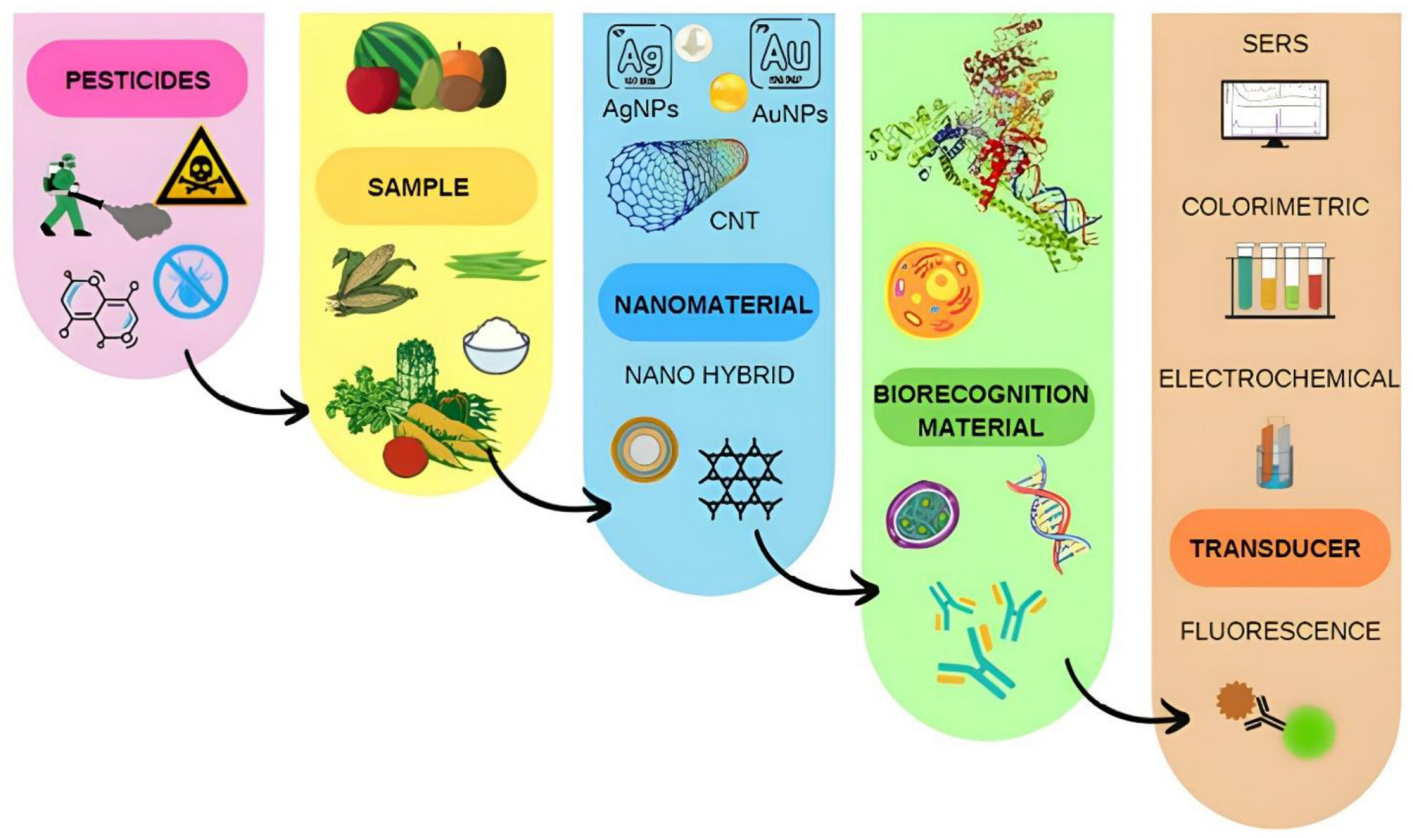
Schematic diagram of fabrication of biosensor. The figure is a part of free to use, creative commons license and requires no copyright permission.

Phycotoxins intoxicate humans causing digestive and neurological disorders, as well as respiratory stress, skin problems and prove to be fatal during the lifetime (72). CNTs has been used previously in mice models in the healthcare sector for studying various processes (73). Shafiq et al. (19) studied better ways to detect palytoxin and microcysin-LR utilizing immunoassays and electro-chemiluminiscence properties of MWCNT and SWCNT. Currently electro chemical biosensors synthesized via novel nanomaterials, such as Boron CNTs (single and multi-walled) are utilized in the identification of toxins in food. Zhang et al. synthesized a novel biosensor for the identification of cyanide in *Prunus armeniaca*. In another research, CNTs and other nanomaterials were utilized by transforming the CNTs into functionalized c-CNTs for field applications (22). For the detection of biochemical contaminants within food as explained by Girigoswami et al. (74), various technologies have been devised that help in overcoming issues related to food biosafety, using nanomaterial-based biosensors. These techniques are efficient in solving various hurdles in the field of biosensing and result in better, feasible and faster results as compared to their counterpart techniques. All the techniques used in the research were based on the principles of electrochemical sensing and fluorescent signaling respectively (74). In another research, H_2_O_2_ was detected by ferrocene-grafted carbon nanotube in a non-enzymatic manner by using an electrochemical process (75). The foregoing accounts make it clear that CNTs can be used as efficient tools in biosensing toxins and prove to be useful in food safety applications in the coming future (76). Figure 5 Illustrates the manufacturing steps regarding biosensor fabrication.

### 4.2 Chemicals and pesticides

Serious health risks are linked with chemical exposure from the environment (77). The risk of chronic diseases such as neurodegenerative diseases, diabetes and cancer along with decreased fertility and birth defects are all linked to exposure to different pesticides (77, 78). The detection of these types of contaminants and their estimation is very crucial for the safety of people (23, 79). Owing to their excellent adsorption properties, CNTs have been utilized for isolation and extraction of pesticides from different food samples, with help of various techniques including the application of Solid-Phase Extraction (SPE) and Solid-Phase Micro-Extraction (SPME) (19). The detection of compounds is linked to the extraction techniques used for their quantification. These techniques include High performance liquid chromatography (HPLC), Liquid chromatography (LC), and Gas chromatography (GC). The use of CNTs is permissible owing to their cheap and effective methods of fabrication, thus allowing the detection of pesticides with 15-folds of sensitivity as compared to counterpart techniques (80). In research, c-MWCNTs gave better sensitivity values regarding biosensing mechanisms. In a research Chen et al. (29) employed MWCNTs to synthesize a acetylcholinesterase (AChE)-based electrochemical sensor for feasible and effective pesticide detection in food samples. These processes are very important since they allow the detection of toxins and pesticides with high sensitivity (81, 82). This useful trait regarding CNTs can prove to be useful in future bioremediation applications and treatment of soil and water.

### 4.3 Pathogens

CNTs have been employed in medical applications for the detection of pathogens including *Escherichia coli* and *Salmonella*, which can be detected through the application of SWCNT based fluorescence microscopy (19). *E. coli* is a gram-negative bacterium which poses great threat to humans and causes a range of ailments. *E. coli* encompasses galactose-binding surface proteins that have strong interactions that can be detected by Galactose single-walled nanotubes (Gal-SWNTs) (3). Real-time detection of bacteria can be also be done with CNT-based sensors in which MWCNT can be used for the screening of *Enterobacter cloacae* by assay methods (83). SWCNT-based immunosensor working on the principle of electrochemical impedance is utilized in onsite identification of *Listeria monocytogenes*, which is a pathogenic bacterial strain and causes many diseases in living things (84). SWCNTs use covalent bonding to detect *Salmonella* via biosensing mechanism. In the process of detection, firstly, the ssDNA probe solution at room temperature was incubated on electrodes for about 2 h. The sensor exhibited a concentration in the typical range of 1 × 10^−9^ mol/L of DNA sensitivity. In another experiment *Salmonella* was detected using PCR but using deposition mechanism of ITO (indium tin oxide) to MWCNTs which resulted in amino-modified apt sensor process. Researchers were able to detect 148 bp invA gene expressed in both strains of *Salmonella* within a limit of 103 cfu-m. In another experiment, SWCNTs-based nanosensor was utilized for the detection of strains coupled with anti-*E. coli* antibodies and found the limit to be 102 cfu-m. Later researchers verified the process by with *Staphylococcus aureus* that gave negligible variation in the signal thus confirming the sensitivity and specificity results regarding *E. coli* (85). This phenomenon shows the importance of CNTs in the detection of pathogenic strains which can be useful in food protection processes and health safety, in the future.

### 4.4 Preservatives and food dyes

**Figure 6:**
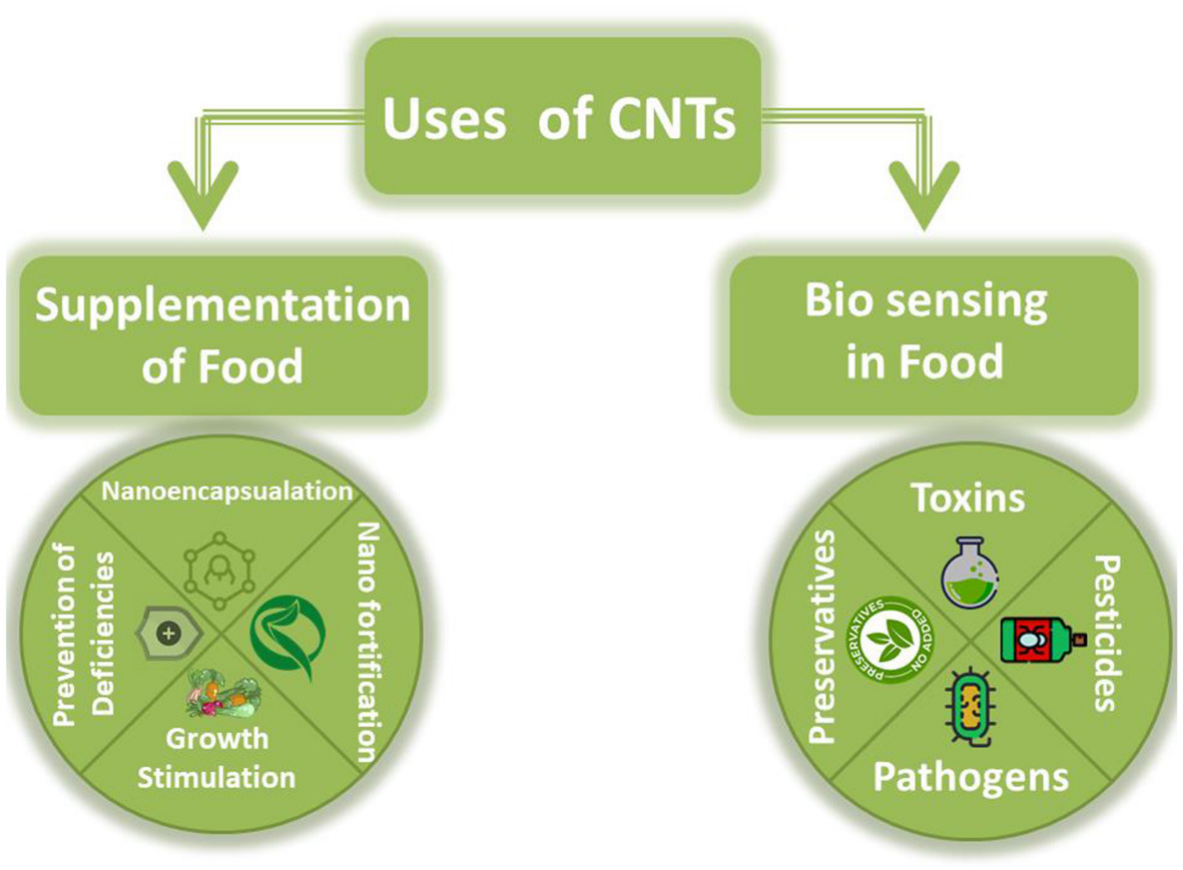
Applications of CNTs as food supplements and nanobiosensors.

Food colorings and preservatives are considered toxic, when utilized beyond permissible dose limits. In a research, Ionic-liquid nanomaterials conjugated with MWCNTs were used to detect a red colored carcinogenic dye known as Sudan-I in chili powder and sauce. They can also be used to detect sunset yellow and tartrazine which can pose health threats when consumed in higher quantities. Similarly, nanoparticles of zinc oxide and nanocomposites conjugated with CNTs have been used to detect sudan and bisphenol A (hazardous molecule expelled from containers of plastic) (19). Functionalized CNTs contain oxide nanoflowers which helps in ultra-sensitive identification of preservatives in food including tert-butylhydroquinone, by utilizing 3D copper (86). Some innovative techniques based upon field effect transistor (FET) and voltammetry (cyclic) utilized MWCNTs and SWCNTs coupled with various nanomaterials for the detection of metals including Cd ions, and various dyes like tartrazine, sunset yellow, and Bisphenol A. In case of Sudan 1, HPLC was also used along with CNTs treatment (19). MWCNTs coupled with tyrosine have been used as nanoadsorbents to expel the toxic additive coloring known as methylene blue (87). This research shows the ability of CNTs to be utilized as detective materials and their potential to be used in food applications and safety, respectively (88, 89). Figure 6 illustrates the applications of CNTs as described in this review.

## 5 Global market scenario and commercial products

CNTs have been utilized in a variety of applications ranging from biological substances to mechanical products. This portion refers to the use of CNTs in various areas and in the production of a number of substances. Due to their beneficial thermodynamic, electrical and chemical properties, CNTs have gained an important place in various economic sectors (47). The tensile strength and conductivity parameters allow CNTs to be used in the fabrication of a range of products (90). Table 2 illustrates some commercial products and companies that have utilized CNTs in the production of a range of products. The importance of CNTs can be observed from the fact that almost all niches of industrial sectors have used CNTs in products fabrication and enhancement of quality parameters.

**Table 2:** CNT products developing and selling companies.

**S. No**.	**Company**	**Products and application**	**URL**
1	Aneeve	Composites, electronic circuits, diagnostics, FET applications	http://aneeve.com/
2	ANS	Composites, synthetic fibers, battery electrodes	http://www.appliednanostructuredsolutions.com/archives/4
3	BlueNano	Energy products, CNTs based powders, battery electrodes	http://www.bluenanoinc.com/nanomaterials/carbon-nanomaterials.html
4	Eikos	Energy products, coatings, conductors	http://www.eikos.com/
5	Intel	Microelectronic circuits, switches, FET	http://www.intel.com/
6	NanOasis	Energy products, filtration membranes	http://www.nanoasisinc.fogcitydesign.com/
7	NanoIntegris	Coatings, conductors, FET, diagnostics	http://www.nanointegris.com/
8	Nanomix	Biotechnological products, sensing and diagnostic applications	http://www.nano.com/
9	Seldon	Energy products, water purification systems	http://seldontechnologies.com/
10	Toray	Coatings, conductors, thermal sensing applications	http://www.toray.com/

Adapted from De Volder et al. (91).

## 6 Conclusion

CNTs possess various useful properties due to their large surface area, electrical and mechanical properties. CNTs are very important in the field of food biotechnology where they play pivotal role in the provision of food supplements and vital role in biosensors applications for identification and quantification of contagions food products. They are actively being used for the detection of preservatives, pathogens, toxins, food dyes, chemicals and pesticides and have the potential to be used as suitable candidates in dairy industry. Although, being in the stage of development, many nanomaterials possessing CNTs have been reported with promising results. The domain itself is new and there have been claims of nanomaterials and CNTs and their association with health complications. The claims revolve around the concept of CNTs being unpredictable due to their smaller size. Further studies on the biosafety regarding CNTs need to be conducted in the future to ensure their safe use in different applications. Overall, CNTs possess great potential, possibly being an integral part of the future technology in the food industry and related applications. The use of CNTs are far superior as compared to their drawbacks but a comprehensive research needs to be shaped in understanding their true potential and role.

## 7 Prospects and challenges

The future holds a promising market for CNT based products for quality control of food products. Nanobarcodes have emerged as a new concept which can be assigned for product authenticity. Protection from allergens and pathogens can be assured due to the physical properties of CNTs including inhibition of biofilms. Nanopackaging and nanoprocessing can be utilized to increase the shelf life of food items. Currently there are many challenges and complications faced when dealing with CNTs. These include (a) determination of concentration levels of CNTs, (b) toxicity problems that they may pose if the concentration is above the permissible limits, (c) cascade of disruptive events they can be caused due to their highly reactive nature, and (d) handling due to small size which needs professional care. These challenges can be addressed via rigorous research and new avenues can be brought forward regarding the use of CNTs in different applications.

## Author contributions

MM: Conceptualization, Data curation, Investigation, Writing—original draft. TA: Data curation, Writing—review & editing. NS: Funding acquisition, Writing—review & editing. ME-S: Supervision, Writing—review & editing. HE-N: Validation, Visualization, Writing—review & editing.
